# They Keep Their Hands Clean

**Published:** 1897-06

**Authors:** 


### They Keep Their Hands Clean.

There are a great many good results which accompany a full understanding of the germ theory. One of these is that the doctor is taught to wash his hands! Some of our readers may have noticed occasionally (during their professional career) that now and then a physician has finger nails as black as Mark Twain's cat in a dark cellar. But now that all physicians are likely to perform some kind of an operation which demands thorough asepsis, at least once a month, we are assured that these accumulations of time will be disturbed from their years of repose.

